# Hepatocyte growth factor signalizes peritoneal membrane failure in peritoneal dialysis

**DOI:** 10.1186/1471-2369-15-201

**Published:** 2014-12-17

**Authors:** Ana Paula Bernardo, José C Oliveira, Olívia Santos, Maria J Carvalho, António Cabrita, Anabela Rodrigues

**Affiliations:** Nephrology Department, St. António Hospital - Oporto Hospital Center, Oporto, Portugal; Clinical Pathology, St. António Hospital - Oporto Hospital Center, Oporto, Portugal; UMIB/ICBAS/UP, Oporto, Portugal; Largo Prof. Abel Salazar, 4099-001 Porto, Portugal

**Keywords:** Hepatocyte growth factor, Peritoneal membrane, Ultrafiltration failure, Water transport

## Abstract

**Background:**

Hepatocyte growth factor (HGF) counteracts peritoneal fibrosis in animal models and in-vitro studies, but no study explored effluent HGF in peritoneal dialysis (PD) patients with ultrafiltration failure (UFF). Our aim was to assess the relationship between effluent HGF with UF profile, free water transport (FWT) and small-solute transport.

**Methods:**

We performed 4-hour, 3.86% PET with additional UF measurement at 60 minutes in 68 PD patients. MTAC_creatinine,_ FWT, small-pore ultrafiltration, and effluent HGF were quantified.

**Results:**

Effluent HGF negatively correlated with UF (r = −0.80, p = 0.009) and FWT (r = −0.69, p = 0.04). Patients with UFF had higher dialysate HGF (103 pg/mL vs 77 pg/mL, p = 0.018) and, although not statistically significant, those with FWT compromise had also higher dialysate HGF compared with subgroup of UFF without FWT compromise (104 pg/mL vs 88 pg/mL, p = 0.08). FWT ≤ 45% without clinical UFF was documented in some patients who also had increased effluent HGF.

**Conclusions:**

Dialysate HGF concentration is significantly higher among patients with UFF, specially, if FWT is impaired, being a sign of peritoneal membrane deterioration.

## Background

Ultrafiltration failure (UFF) is still a challenging complication of peritoneal dialysis and its prevalence increases with time on PD [1–3]. In long-term patients, UFF is more severe and often associated with free water transport (FWT) compromise [4–6]. The two-in-one peritoneal equilibration test allows simultaneous quantification of FWT and small pore ultrafiltration, being a simple method for timely detection of membrane failure, as we previously reported [7]. Since it is known that increased submesothelial fibrosis is an early and progressive lesion ultimately associated with UFF [8], the search for an effluent marker related to membrane fibrosis process and exhibiting a good correlation with ultrafiltration and FWT would be clinically important. Such a marker could timely detect peritoneal membrane failure. Besides it should be desirable that such marker could signalize peritoneal membrane deterioration even before clinically relevant UFF.

Hepatocyte growth factor (HGF) is known to play a crucial role in the repairing process of tissues and preventing organ fibrosis [9–12]. Yu et al. demonstrated, for the first time, that human peritoneal mesothelial cells constitutively synthesized HGF [13], and that treatment of human peritoneal mesothelial cells with HGF blocks high glucose-induced epithelial-to-mesenchymal transition (EMT). More recently, Ueno T, et al. showed that HGF secreted by mesenchymal stem cells was implicated in the inhibition of the transforming growth factor β1 signaling and ameliorated peritoneal fibrosis in an **ex-vivo** study [12]. It is thus relevant to increase the knowledge on HGF clinical value, in patients under PD, as it may possibly point to new diagnostic opportunities and therapeutic avenues. Therefore clinical investigation under this subject is mostly important and needed.

Impaired FWT is assumed to indicate a more severe functional and structural membrane lesion due to aquaporin disfunction or interstitial changes [6, 14–16], but there are no clinical studies exploring the associations between effluent HGF, ultrafiltration failure, FWT and small-solute transport. For that reason, we performed a clinical investigation in order to assess, in a prevalent PD population, the relation between dialysate HGF and the ultrafiltration profile, FWT quantification, and small-solute transport.

## Methods

### Patients and procedures

This cross sectional study enrolled 68 patients of our Unit. All the patients performed a 4-hour, 3.86% glucose modified peritoneal equilibration test (PET) with total temporary drainage at 60 minutes [“Two-in-one” protocol, as published before [7]]. This protocol allows free water transport quantification, beyond a simple calculation of sodium sieving [7]. None of the patients had peritonitis during the study or the preceding 6 weeks. During the procedure, we used PD solutions low in glucose degradation products, according to the individual patient’s prescription. The volume of dialysis solution was determined by weight, without flushing the system and before filling the peritoneum. Blood and dialysate samples (each approximately 10 mL) were taken at instillation of the dialysate and after 60 and 240 minutes. At 60 minutes, we performed an additional measurement of UF by total drainage of the peritoneal cavity. This drained volume was weighed and then immediately reinfused. Finally, after 240 minutes, the peritoneal cavity was drained and the volume obtained was weighed.

PETs with an ultrafiltered volume ≤ 400 mL/4 h were considered to represent ultrafiltration failure (UFF).

All patients provided written informed consent for participation, and the study was approved by the Ethics Committee of St. António Hospital – Oporto Hospital Center.

### Measurements

Creatinine and sodium were measured both in plasma and dialysate. For creatinine, the Jaffé compensated method was used. The dialysate creatinine concentration was corrected for interference by glucose according to our laboratory standards. Sodium was measured using indirect ion-selective electrodes. Effluent samples taken at 4 hour were immediately stored at −70°C, until they were used to measure HGF, VEGF and CA125. Effluent CA125 was determined with an electrochemiluminescence method on an automated analyzer (COBAS e-411, Roche Diagnostics GmbH). Effluent HGF and VEGF levels were determined by ELISA technique according to the manufacturer’s instructions (IBL – Immuno-Biological Laboratories Co. Ltd). The intra and inter-assay variations were 8,8%% and 10,0%, respectively for HGF and 5,9%, and 9,4% for VEGF. The sensitivity was 11 pg/mL for HGF and 1 pg/mL for VEGF. Both the assays are considered highly specific for the cytokines, and no significant cross-reactivity was observed.

### Calculations

Patients were characterized by peritoneal transport status as described by Twardowski et al. [17]. The MTAC_creatinine_ was calculated by the simplified Garred model [18].

FWT and UF through the small pores (SPUF) at 60 minutes were calculated as we previously described [7]. Using a simple algorithm, we also performed a correction for FWT as described by Venturoli and Rippe [19].

### Statistical analysis

Except for time on PD, HGF, HGF/CA125 and VEGF/CA125, all variables had normal distribution. Results are expressed as mean ± SD or as median and interquartile range.

Pearson correlation analysis was used in order to explore possible relations between HGF (with logarithmic transformation) and ultrafiltration profile, FWT and small-solute transport.

For comparison of small solute transport, water transport pathways and effluent markers between patients with and without ultrafiltration failure, Mann–Whitney U test was used.

In order to study our patients ultrafiltration failure profile we made a comparison of small solute transport, water transport pathways and effluent markers between patients with FWT ≤ 45% and D/P_Creatinine_ ≥ 0.81 and patients without FWT compromise and non-fast transport category, using Mann–Whitney U test.

Unpaired Student t-Test or Mann Whitney U-test to compare patients with FWT ≤ 45% and FWT > 45%, as appropriate, according to the variables involved.

## Results

### Solute, fluid transport parameters and concentration of cytokines in dialysate

Table 1 summarizes the peritoneal transport characteristics evaluated with a combined (“two-in-one”) PET performed in 68 study patients [35 men; mean age: 50 ± 14 years; 14 patients were diabetic; 16 were anuric; 36 were on APD; PD vintage 18.7 ± 23.5 months (range 1 – 121 months)]. According to small solute transport characteristics, 1 (1.5%) patient was classified as slow transporter (D/P_Creatinine_ ≤ 0,49), 10 (14.7%) as slow-average (0,50 ≤ D/P_Creatinine_ ≤ 0.64), 41 (60.3%) as fast-average (0,65 ≤ D/P_Creatinine_ ≤ 0.80), and 16 (23.5%) as fast transporters (D/P_Creatinine_ ≥ 0,81). Concerning water transport pathways, FWT accounted for 37.15% of the UF at 60 minutes, and once corrected (for sodium diffusion, cumulative UF volume through the large pores, and cumulative lymphatic absorption at 60 minutes), its contribution increased to a mean value of 45.46%.

**Table 1:** **Peritoneal transport characteristics and effluent cytokines in 68 stable patients assessed during a 4-hour, 3.86% glucose peritoneal equilibration test with temporary drainage at 60 minutes**

Variable	Mean ± SD Median (IQR 25%-75%)
**Total UF at 4 h (mL)**	669,12 ± 226,76
**SPUF (mL)**	323,36 ± 129,34
**FWT (mL)**	183,26 ± 63,02
**FWT**_**corrected**_ **(mL)**	224,12 ± 66,51
**%FWT**	37,15 ± 11,79
**%FWT**_**corrected**_	45,46 ± 11,11
**D/P**_**Creatinine**_	0,76 ± 0,12
**MTAC**_**Creatinine**_ **(mL/min)**	11,32 ± 7,14
**Effluent HGF (pg/mL)**	77,17 (68,83 – 94,31)
**Effluent VEGF (pg/mL)**	13,96 ± 4,92
**CA125 (U/mL)**	19,57 ± 11,31

SD = standard deviation; UF = ultrafiltration; SPUF = UF through the small pores at 60 minutes; FWT = free water transport at 60 minutes; FWT_corrected_ = FWT with an algorithm correction according to Venturoli and Rippe [16]; MTAC = mass transfer area coefficient; D/P = dialysate-to-plasma ratio; HGF = hepatocyte growth factor; VEGF = vascular endothelial growth factor; CA 125 = cancer antigen 125.

### HGF correlations with ultrafiltration, FWT and small-solute transport

**Figure 1:**
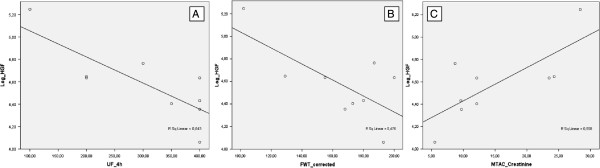
**HGF correlations with ultrafiltration, FWT and small-solute transport. (A)** Correlation between effluent hepatocyte growth factor (HGF) and total ultrafiltration at a 4 h, 3.86% glucose PET (UF240), in patients with ultrafiltration failure (Pearson r = −0.802, p = 0.009); **(B)** correlation between effluent hepatocyte growth factor (HGF) and FWT corrected according to Venturoli and Rippe [16], in patients with ultrafiltration failure (Pearson r = −0.690, p =0.04); **(C)** correlation between effluent hepatocyte growth factor (HGF) and creatinine mass transfer area coefficient (MTAC_creatinine_), in patients with ultrafiltration failure (Pearson r = 0.747, p = 0.021).

HGF measured in the effluent significantly correlated with total ultrafiltration at a 4 h, 3.86% glucose PET (r = −0.358, p = 0.003), with FWT (r = −0.407, p = 0.001) and MTAC_creatinine_ (r = 0.355, p = 0.003). These correlations were even stronger when we focused the analysis in patients with ultrafiltration failure (Figures 1A,B,C). In those patients, HGF exhibited a strong negative correlation with total ultrafiltration and FWT (r = −0.802, p = 0.009 and r = −0.690, p = 0.04, respectively) and a strong positive correlation with MTAC_creatinine_ (r = 0.747, p = 0.021). No correlation was found between dialysate HGF concentration and small pore water transport (neither in global population, nor in the UFF group).

### Patients with ultrafiltration failure compared with stable patients

Among the 68 study patients, 9 (13.2%) had UFF (total UF ≤ 400 mL/4 h). Although not statistically significant, patients with UFF had been on PD for a longer time, had higher D/P_creatinine_ and MTAC_creatinine_(Table 2). HGF concentration was significant higher in patients with UFF (median 103.0 pg/mL IQR [79.8–110.8]) compared with stable patients (median 77.1 pg/mL IQR [68.1–92.6], p = 0.018). Although not statistically significant, patients with UFF had also a higher ratio HGF/CA125 (Table 2).

**Table 2:** **Comparison of small solute transport, water transport pathways and effluent markers between patients with and without ultrafiltration failure**

	Patients with UFF Mean ± SD Median (IQR 25-75%)	Patients without UFF Mean ± SD Median (IQR 25-75%)
Patients (n)	9	59
Time on PD (months)	6.0 (4.0 – 35.5)	7.0 (4.0 – 27.0)
Total UF at 4 hour (mL)	305.56 ± 113.04^a^	724.58 ± 184.38^a^
SPUF (mL)	192.89 ± 59.41^a^	343.27 ± 125.68^a^
FWT (mL)	134.89 ± 35.07^b^	190.64 ± 63.25^b^
FWT_corrected_(mL)	165.22 ± 31.93^c^	233.10 ± 65.95^c^
%FWT	41.87 ± 13.07	36.43 ± 11. 53
%FWT_corrected_	51.16 ± 12.29	44.59 ± 10.77
D/P _Creatinine_	0.81 ± 0.12	0.75 ± 0.11
MTAC _Creatinine_ (mL/min)	14.88 ± 8.23	10.78 ± 6.87
Effluent HGF (pg/mL)	103.01 (79.83 – 110.78)^d^	77.07 (68.05 – 92.58)^d^
Effluent VEGF (pg/mL)	15.10 ± 5.68	13.79 ± 4.82
CA125 (U/mL)	24.63 ± 19.63	18.89 ± 9.75
HGF/CA125 (pg/U)	6.22 (2.28 – 8.27)	4.64 (3.30 – 6.36)
VEGF/CA125 (pg/U)	0.70 (0.53 – 1.39)	0.76 (0.54 – 1.08)

SD = standard deviation; UF = ultrafiltration; SPUF = UF through the small pores at 60 minutes; FWT = free water transport at 60 minutes; FWT_corrected_ = FWT with an algorithm correction according to Venturoli and Rippe [16]; MTAC = mass transfer area coefficient; D/P = dialysate-to-plasma ratio; HGF = hepatocyte growth factor; VEGF = vascular endothelial growth factor; CA 125 = cancer antigen 125.

^a^Mann–Whitney U test p <0.0001, comparing patients with and without ultrafiltration failure.

^b^Mann–Whitney U test p = 0.006, comparing patients with and without ultrafiltration failure.

^c^Mann–Whitney U test p = 0.002, comparing patients with and without ultrafiltration failure.

^d^Mann–Whitney U test p = 0.018, comparing patients with and without ultrafiltration failure.

### Ultrafiltration failure profile

**Figure 2:**
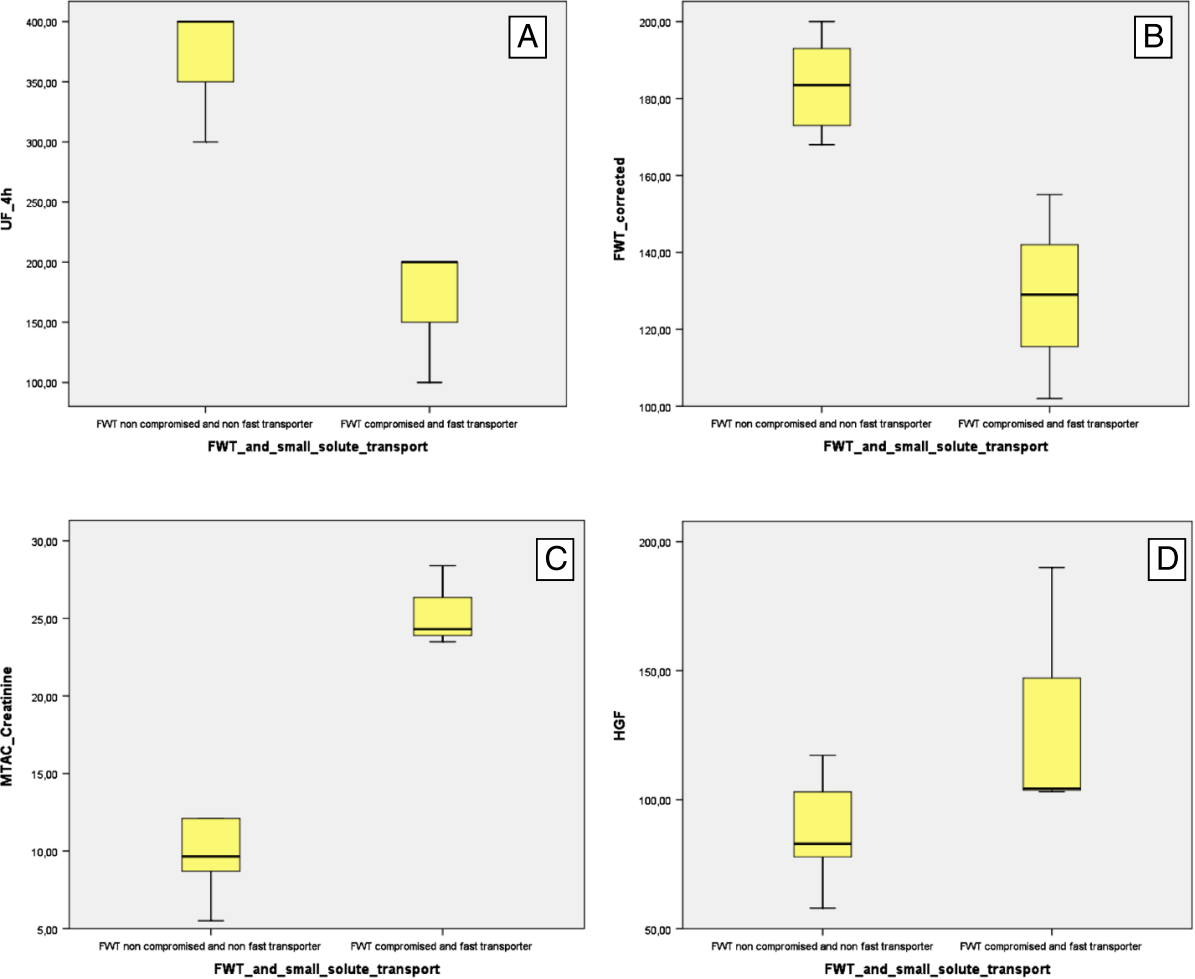
**Ultrafiltration failure profile.** Comparison within patients with UFF, between 6 patients with non compromised FWT and non fast transporters versus 3 patients with FWT ≤ 45% and fast transporters: (A) Total ultrafiltration at a 4 h, 3.86% glucose PET (375.0 ± 41.83 mL vs 166.67 ± 57.74 mL, p = 0.024). **(B)** FWT quantification at 60 minutes in a 4 h, 3.86% glucose PET (183.5 ± 12.14 mL vs 128.67 ± 26.50 mL, p = 0.024) **(** **C** **)** MTAC_creatinine_ (9.62 ± 2.45 mL/min vs 25.40 ± 2.63 mL/min, p = 0.024). **(** **D** **)** Effluent Hepatocyte Growth Factor (88.94 pg/mL vs 104.3 pg/mL, p = 0.085). Box plot representation: 75% percentile, 25% percentile, median, and maximum and minimum values.

From the 9 patients with UFF, 3 had a more severe profile characterized by FWT compromise (FWT ≤45%) and increased (D/P_creatinine_ ≥ 0,81). Those patients had significant lower ultrafiltration volume at a 4 h PET (166.7 ± 57.4 mL vs 375.0 ± 41.8 mL, p = 0.024), lower FWT quantification (128.67 ± 26.50 mL vs 183.5 ± 12.14 mL, p = 0.024) and higher MTAC_creatinine_ (25.40 ± 2.63 mL/min vs 9.62 ± 2.45 mL/min, p = 0.024) (Figure 2A,B,C). Although not statistically significant, patients with the more severe UFF profile had also higher values of HGF measured in the effluent (104.3 pg/mL vs 88.94 pg/mL, p = 0.085) (Figure 2D).

Tree other patients showed a less severe UFF profile, with increased effective capillary surface but preserved FWT (>45%). And still 3 incident patients (3 – 6 months on PD), were average transporters with preserved FWT, in whom higher lymphatic absorption was by exclusion presumed.

### FWT profile in patients without UFF

From the 59 studied patients without UFF, 33 had a FWT ≤45%. Those patients had a mean UF volume at 4 h PET equivalent to patients with FWT > 45% (Table 3). Although not statistically different, patients with FWT ≤ 45% had higher effluent HGF, when compared with patients with FWT > 45%. In spite of higher effluent HGF concentration, the HGF/CA125 ratio was significantly lower in patients without UFF and FWT ≤45% compared with patients without UFF and FWT >45% (3.65 IQR [ 2.96-5.60] vs 5.19 IQR 4.02-8.37], p = 0.014).

**Table 3:** **FWT profile in patients without UFF: comparison of small solute transport, water transport pathways and effluent markers between patients with FWT ≤ 45% and FWT > 45%**

	Patients without ultrafiltration failure	
	Patients with FWT ≤ 45% Mean ± SD Median (IQR 25-75%)	Patients with FWT > 45% Mean ± SD Median (IQR 25-75%)
Patients (n)	33	26
Time on PD (months)	6.0 (4.0 – 19.0)	8.5 (5.0 – 30.25)
Total UF at 4 hour (mL)	739.39 ± 210.56	705.77 ± 146.51
SPUF (mL)	412.9 ± 108.54^a^	254.81 ± 83.74^a^
FWT (mL)	173.42 ± 60.43^b^	212.50 ± 60.98^b^
FWT_corrected_(mL)	221.51 ± 66.11	247.81 ± 63.97
%FWT	29.10 ± 6.50^a^	45.74 ± 9. 66^a^
%FWT_corrected_	37.56 ± 5.62^a^	53.52 ± 8.96^a^
D/P _Creatinine_	0.76 ± 0.08	0.73 ± 0.14
MTAC _Creatinine_ (mL/min)	10.45 ± 5.14	11.20 ± 8.69
Effluent HGF (pg/mL)	77.17 (68.42 – 93.89)	75.89 (66.47 – 86.51)
Effluent VEGF (pg/mL)	14.19 ± 4.96	13.28 ± 4.68
CA125 (U/mL)	22.28 ± 10.90^c^	14.56 ± 5.82^c^
HGF/CA125 (pg/U)	3.65 (2.96 – 5.60) ^d^	5.19 (4.02 – 8.37) ^d^
VEGF/CA125 (pg/U)	0.68 (0.51 – 0.90)	0.89 (0.67 – 1.29)

Variables presented as mean ± SD or median (interquartile range) accordingly. SD = standard deviation; UF = ultrafiltration; SPUF = UF through the small pores at 60 minutes; FWT = free water transport at 60 minutes; FWT_corrected_ = FWT with an algorithm correction according to Venturoli and Rippe [16]; MTAC = mass transfer area coefficient; D/P = dialysate-to-plasma ratio; HGF = hepatocyte growth factor; VEGF = vascular endothelial growth factor; CA 125 = cancer antigen 125.

a) T-test p <0.0001, comparing patients with FWT ≤ 45% and FWT > 45%.

b) T-test p = 0.017, comparing patients with FWT ≤ 45% and FWT > 45%.

c) T-test p = 0.001, comparing patients with FWT ≤ 45% and FWT > 45%.

d) Mann–Whitney U test p = 0.014, comparing patients with FWT ≤ 45% and FWT > 45%.

## Discussion

To our knowledge, the present study is the first to report a clinical significant relation between HGF measured in the effluent and ultrafiltration profile, FWT quantification and small-solute transport in peritoneal dialysis patients. Since HGF is involved in the process of peritoneal submesothelial fibrosis [12, 13], this study also suggest some structural-functional correlations.

Although impaired FWT is frequently associated to aquaporin dysfunction, several studies provided indirect evidence that FWT can also be impaired in situations of decreased peritoneal water permeability due to interstitial changes, especially in long term patients [6]. Simulations of osmotic ultrafiltration failure in CAPD using a serial three-pore membrane/fiber matrix model [14] documented the uncoupling of small solute transport from LpS in computer simulations of UFF, whenever changes both in vasculature and in interstitium are taken into account, supporting further the role of the peritoneal membrane interstitium in fluid transport. Moreover, Devuyst and Rippe stated in a recent review [16] that reduced LpS in long-term PD has been attributed to reductions in AQP1-meditated water transport, but it might also be the result of a combination of increased vascularization and fibrotic scar tissue in the peritoneum.

When we analyze the reasons for UFF in our study we distinguish 3 groups of patients. Three had a more severe profile characterized by FWT compromise (FWT ≤45%) on top of increased effective capillary surface, also with higher values of effluent HGF. Three patients presented an increased effective capillary surface but FWT was not impaired. Another 3 incident patients were average transporters with normal FWT. A high effective lymphatic absorption rate could be the reason for UFF in those incident patients since high lymphatic absorption is recognized to be mostly a cause of inherent UFF [5]. This variability in UFF patterns that we found was also documented by Waniewsky et al. [15] that recognized that UFF due to high peritoneal absorption could be associated with normal or decrease fractional contribution by transcellular pores to hydraulic conductivity. It is noteworthy that mean HGF in those 3 patients (with a functional UFF) was significantly lower compared with the 3 patients that had impaired FWT [77.84 (67.9 – 79.8) vs 104.3 (103.7 – 147.16), p = 0.05].

In contrast with previous studies [4, 6, 20], MTAC_creatinine_ was higher but not statistically different between patients with and without UFF. This is due to the presence of many incident fast transporters (mean time on PD 6 ± 4 months) in the non UFF group.

Report and interpretation of causes of UFF as well as absolute and fractional FWT may vary according to the PD vintage. Our results are in accordance with Parikova et al., [6] that documented an early stage UFF associated with decrease of absolute FWT dependent on increased effective capillary surface without significant decrease of FWT contribution, while later the loss of osmotic conductance to glucose lead to a significant decrease of FWT fraction. In fact, all 9 patients with UFF had a significant decrease in absolute FWT compared with patients without UFF, but only 3 had a reduction in FWT contribution to 60 minutes ultrafiltration.

Moreover, our study adds further clinical evidence to the recent report from Nakamura S. et al. [21]. Those authors studied focal HGF expression in peritoneum biopsies of a small number of peritoneal dialysis patients, with and without UFF. Although they did not measure dialysate HGF concentration, they demonstrated an increased expression of HGF in peritoneal tissues of CAPD patients with low ultrafiltration capacity compared with those with a normal ultrafiltration profile. Given the already mentioned protective effects of HGF on peritoneal fibrosis [11–13], we hypothesized that the increased peritoneal HGF expression demonstrated by Nakamura S. et al. [21], and the higher dialysate HGF concentration that we found in our patients with UFF, can be seen as a reactive mechanism to peritoneal membrane lesion. This is also supported by the fact that patients with more severe forms of UFF (with FWT compromise besides an increase in small-solute transport), presented higher dialysate HGF concentration.

The effluent HGF concentration on patients under PD was addressed, until now, by one single clinical study [22]. However, Mizuiri S et al. [22], only compared dialysate HGF concentration according to small solute transport status, and found that fast transporters had higher effluent HGF concentration compared with others small solute transport categories, which we also reproduce (data not shown). Unfortunately such study gave no information about UFF or water transport pathways quantification.

As others [23] we also found significantly higher dialysate VEGF concentration in fast transporters compared with non-fast transporters (data not shown). Although it remains uncertain if this increase in VEGF production corresponds to an increased production of intraperitoneal vasoactive substances, as might occur in incident fast transporters, or if it is the result of epithelial-to-mesenchymal transition, dialysate VEGF was not discriminative of UFF. On the contrary, effluent HGF was informative not only about UFF but also highlighted more severe UFF profile, with FWT compromise.

We are aware that the mass of mesothelial cells could affect the levels of intraperitoneal growth factors in PD patients. Although there are some controversies about the use of CA125 as an index of mesothelial cells mass or their functional properties [24–26], we also documented a higher HGF/CA125 ratio in UFF patients compared with stable patients, indicating a reactive increased HGF production beyond that we would expect for the mesothelial cell mass.

Since there are no clinical studies that had examined plasma and effluent HGF, and we found a correlation with small-solute transport, we might question whether effluent HGF concentration could depend on plasma HGF levels. We think that this is not plausible for various reasons. First, the peritoneal permeability is expected to be poor, since HGF is a heterodimeric molecule composed of a 69 KDa alpha subunit and a 34 KDa beta subunit. Second and mostly important, human peritoneal cells constitutively synthesized HGF [13]. For these reasons, we believe that the HGF protein detected in the effluent is locally produced.

The use of effluent biomarkers as an early sign of peritoneal membrane alterations is currently under debate [27–29] specially because clinical factors cannot give an accurate individual prediction for EPS [30].

In a very recent report [27], MCP-1, IL-6 and CCL15 were found at higher levels in the dialysate of patients who subsequently developed EPS. However, by logistic regression analysis, these cytokines did not improve prediction of future EPS above known clinical factors, as PD vintage and peritoneal small solute transport. On the contrary, Sampimon et al. [28] concluded that dialysate appearance rate of CA125 and IL-6 combined was potentially useful for an early diagnosis of EPS. None of these studies explored the associations between the cytokines measured in the dialysate and ultrafiltration or water transport pathways in a peritoneal equilibration test. These would be of great importance as we know that in the 2 years that precede an EPS diagnosis, a proportion of patients with EPS present an uncoupling between the membrane ultrafiltration capacity and the peritoneal membrane small solute transport [30]. This fact gives even more strength to the necessity of finding a biomarker that correlates both with ultrafiltration and with water transport pathways, and not only to the membrane small solute transport status. HGF can be easily measured, without specific preparation of the dialysate sample, by commercially available highly specific assay (IBL-Immuno-Biological Laboratories Co.Ltd), with acceptable inter-assay variability (10%); it is constitutively synthesized by human peritoneal mesothelial cells, blocks high glucose-induced epithelial-to-mesenchymal transition (EMT) and was implicated in the inhibition of the transforming growth factor β1 signaling, ameliorating peritoneal fibrosis in an ex-vivo study [13]. According to our present investigation, dialysate HGF concentration increases as ultrafiltration decreases in a 4-hour, 3,86% glucose PET. The fact that dialysate HGF concentration is even higher among patients with FWT compromise and fast transport status increases the likelihood of effluent HGF concentration being related with peritoneal deterioration, as a reactive repairing mechanism, and not with a functional characteristic, as for example an higher effective lymphatic absorption rate, hardly measurable in clinic.

In this study we reported a mean FWT_corrected_ fraction of 45%, which is in line with our previous studies [7], and with the FWT fraction reported by La Milia [31]. A FWT ≤ 45% is clinical relevant in patients with UFF, since it may signalize aquaporin dysfunction or interstitial fibrosis with glucose osmotic conductance compromise, with important repercussions for PD prescription. However, there is no knowledge yet about the clinical value of FWT fraction on patients without UFF. We think that this is another relevant aspect of our investigation: although we did find that patients without UFF but with FWT fraction ≤45% had higher dialysate HGF concentration compared with patients with preserved FWT fraction, the first group had a significant lower mean value of HGF/CA125 ratio compared with the second one. This trend is completely different from that we observed in patients with UFF, in whom we found a higher HGF/CA125 ratio. We hypothesized that this may represent an intermediate level of peritoneal dysfunction, where the patient may already have interstitial changes that lead to a reactive increase in HGF production, while not severe enough to present as UFF. At this point, those patients may still have a preserved mesothelial cell mass which explains the lower HGF/CA125 ratio that we found in this group. Aging, uremia, diabetes are indeed often associated with membrane changes already at PD start possibly justifying in some patients selective FWT compromise in absence of clinically relevant UFF [32–35].

Our study is limited by its cross sectional design and small number of patients with UFF. A longitudinal study is being conducted in order to document the dynamic profile of HGF production and its relationship with peritoneal membrane water transport changes. More studies are needed to increase the knowledge on HGF clinical value, in patients under PD, as it may possibly point to new diagnostic opportunities and therapeutic avenues in the ultrafiltration failure field.

## Conclusions

Since HGF ameliorated peritoneal fibrosis in an **ex-vivo** study [12], a clinical study as ours looks opportune. Our results demonstrated, for the first time, that dialysate HGF concentration is significantly higher among patients with ultrafiltration failure, specially if free water transport is impaired, being an useful marker of progressive peritoneal deterioration.
